# A Cross-Sectional Study of Sexual Health and Well-Being in Married or Cohabitating Middle-Aged and Older Adults in Nouna, Burkina Faso

**DOI:** 10.1007/s10508-025-03223-1

**Published:** 2025-09-30

**Authors:** Oluwadamilola A. Omojola, Cristina A. Osborne, Mamadou Bountogo, Maxime Inghels, Guy Harling, Ali Sie, Dina Goodman Palmer, Till Bärnighausen, Justine I. Davies, Lisa R. Hirschhorn

**Affiliations:** 1 Department of Medical Social Science, Feinberg School of Medicine, Northwestern University, 625 N Michigan Ave 14-013, Chicago, IL 60611 USA; 2 Institute of Applied Health Research, University of Birmingham, Birmingham, UK; 3 Centre de Recherche en Sante de Nouna, Nouna, Burkina Faso; 4 International Institute for Rural Health, University of Lincoln, Lincoln, UK; 5 Institute for Global Health, University College London, London, UK; 6 Heidelberg Institute of Global Health (HIGH), Medical School and University Hospital, Heidelberg University, Heidelberg, Germany

**Keywords:** Sexual activity, Sexual function, Sexual behavior, Middle aged and older adults, Burkina Faso

## Abstract

Sexual health and well-being is important for middle-aged and older adults, but knowledge is limited on prevalence and determinants in countries in Sub-Saharan Africa. We analyzed cross-sectional data collected in 2021 from married or cohabitating adults age ≥ 40 in Nouna, Burkina Faso. Sexual health and well-being were measured using the Sexual Relationships and Activities Questionnaire. Descriptive statistics and multivariable logistic models were used to describe the dimensions of sexual health and well-being—including levels of sexual activity, sexual function, and sexual health concerns—and their associations with age, sex, and education. Among 2336 individuals (56.8% men; mean age 53.2 years) almost all reported having a sexual partner (96.0% men, 93.2% women) in the last year. Men reported more frequent sexual thoughts (71.8% versus 51.2%) and sexual activity (69.4% versus 46.4%, both *p* < 0.001) than women. Being a woman was associated with lower sexual activity (aOR = 0.57) and intercourse frequency (aOR = 0.37, both *p* < 0.001). Older age was associated with reduced sexual thoughts and intercourse (both aOR = 0.93, *p* < 0.001), greater erectile difficulties in men (aOR = 1.06, *p* < 0.001), and arousal difficulties in women (aOR = 1.07, *p* < .001). Higher education was associated with more frequent sexual thoughts (aOR = 1.50, *p* < 0.001), and intercourse (aOR = 1.41, *p* = 0.012). Married and co-habitating middle-aged and older respondents in Nouna reported high rates of sexual activity, with variability in other areas of sexual health and well-being, identifying areas where more research and interventions are needed.

**Supplementary Information:**

The online version contains supplementary material available at 10.1007/s10508-025-03223-1.

## Introduction

There has been a rising interest in sexual health and well-being among middle-aged and older adults, driven by the recognition that sexuality and sexual health are crucial components of overall health (Sinković & Towler, 2019). The widely cited World Health Organization definition of sexual health and well-being includes concepts such as the absence of disease and coercion, respect for sexual rights, and the possibility of sexual pleasure (World Health Organization, n.d.-a). There is also growing evidence on the association of sexual well-being with mental health and quality of life in middle-aged and older people, highlighting the importance of measuring and addressing challenges in this area. Research and policy have identified sexuality as a right for people of all ages and sex (Chepngeno-Langat, 2016) and have demonstrated that sexuality in later life is connected to healthy aging as it has been shown to influence quality of life and perceived life satisfaction (Hardy et al., 2010; Jeste et al., 2013). Although conceptualizations of sexual well-being vary in the literature, it is broadly understood to encompass a biopsychosocial–cultural framework. Syme and colleagues referred to sexual wellness in middle-age and older life within four dimensions: psychological (e.g., cognitions, emotions, and concepts), social (e.g., relationship and shared experience), biological and behavioral (e.g., functioning, behaviors, and scripted sexual activities), and cultural (e.g., age or time in life, and sex and sexual orientation) (Syme et al., 2019).

Despite the growing understanding that sexual well-being is important to people as they age, many surveys do not ask middle-aged or older adults about sexual relationships or activity. These are important elements of sexual health and well-being, and health care providers generally do not recognize the need to assess current function and concerns of this population (Haesler et al., 2016). A steadily aging population implies that a growing number of middle-aged and older individuals will be in romantic relationships and will continue to be sexually active (DeLamater, 2012). A meta-analysis across 29 countries with middle-aged and older adults in aged 40–80 found that sexual interest and desire continues into older age, with more than 80% of men and 65% of women having had sexual intercourse during the past year (Nicolosi et al., 2005). These gaps further highlight the need to understand associations with poor or good sexual well-being in middle-aged and older individuals and identify potential solutions to challenges in sexual health and well-being that emerge or worsen with age (Zhang et al., 2023).

Several biological changes in aging are commonly understood to directly affect sexual health and well-being in older individuals including vaginal dryness, erectile dysfunction, and urogenital atrophy (Morton, 2017). Psychosocial theories of aging go beyond biological aspects and focus on the mental, emotional, social, and cultural challenges of growing old. The literature suggests that long-term partners and long-term romantic or sexual relationships are very important social contexts for middle-aged and older adults (DeLamater, 2012; Kolodziejczak et al., 2019), with well-being shown to be higher among those who are sexually active (Smith et al., 2019). Having a healthy partner is also a protective factor for long-term sexual activity (Bigras et al., 2024). Studies show that most adults over the age of 50 who are in a committed relationship are sexually active, and many remain sexually active up until their 80s (DeLamater, 2012). However, there remain gaps in understanding the prevalence and determinants of sexual health and well-being in middle-aged and older adults (Vasconcelos et al., 2022).

As most research in this population has been conducted in High-Income Countries, this knowledge gap is even greater in Low and Middle-Income Countries (LMICs) including countries in Africa (Chirinda & Zungu, 2016). Studies in Sub-Saharan Africa have found age-related declines in the frequency of sexual activity with distinct sex differences in sexual practices (Agunbiade & Gilbert, 2020; Ede et al., 2023; Nyanzi, 2011; Nyirenda et al., 2025). For example, a descriptive study of sexual satisfaction in 31 Sub-Saharan African countries showed that in both West and East Africa subregions, men were significantly more satisfied with their sex lives than women (Cranney, 2017). There is a need for population-based estimates of sexual health and well-being, in addition to developing validated measures that are culturally and linguistically appropriate. This is of increasing relevance given the aging of populations in many countries in Africa (Duhon et al., 2023).

### Current Study

We analyzed data from a cross-sectional household survey of married or cohabitating individuals aged 40 and above in Nouna, Burkina Faso. Our first aim was to describe sexual health and well-being in this middle-aged and older sub-Saharan African population including sexual behaviors, activities, functioning and satisfaction. Our second aim was to explore the associations between sociodemographic factors, of age, sex, and education, and these sexual outcomes (i.e., sexual behaviors, activities and functioning, sexual health concerns, and partnership satisfaction). For this aim we hypothesized that for middle-aged and older adults in Burkina Faso, the occurrence of sexual activity as well as measures of sexual health functioning and satisfaction would be negatively related to older age. We also hypothesized that sexual activity and positive measures for sexual behaviors and satisfaction would be higher in men and in individuals with higher education. The results will increase knowledge of the prevalence and determinants of sexual health and well-being in this population and guide areas of research and practice in the country and region.

## Method

### Participants

Data were collected as part of the 2021 Centre de Recherche en Santé de Nouna Heidelberg Aging Study (CHAS). The CHAS household survey includes a population-representative sample of people aged 40 and older who live in the Nouna Health and Demographic Surveillance System (HDSS) catchment areas including the town of Nouna and 58 surrounding villages in Boucle du Mouhoun province, north-western Burkina Faso. The predominately rural HDSS site has a total population of approximately 107,000 (Odland et al., 2020). To obtain a representative sample of middle-aged and older adults, a two-part random sample was taken from the Nouna HDSS census in 2015. We allowed for a 25% loss due to mortality, migration, or non-response. In all villages with fewer than 90 adults aged over 40, all adults were selected to take part. In all other villages, a random sample of households with at least one person over 40 years old was taken, and then within each selected household, one age-eligible adult was randomly selected to complete the survey.

Overall, 3089 individuals consented to take part in the household survey, of whom 753 (24.4%) were not eligible for inclusion in the sexual health and well-being questionnaire analysis due to marital status recorded as not married, separated, widowed/ divorced or missing (*n* = 747). An additional six were excluded due to non-completion of the sexual well-being questions. Participants flow is reported in a CONSORT diagram as Fig. S1 in the supplemental materials. In total, 2336 individuals were included for analysis.

### Measures

Collected sociodemographic measures used in this study were age, sex, education (highest level of education completed), and marital status (never married, widowed, separated/divorced, currently married, or cohabiting). Based on consultation with community members in Burkina Faso, the decision was taken by the researchers leading the work during survey design and survey formation to not ask the sexual health and well-being questions of individuals who were not married or cohabitating due to cultural and social acceptability factors as identified by local researchers.

Levels of sexual health and well-being include specific questions on sexual activity, sexual functioning, and concerns about sexual health and well-being were assessed among married or cohabitating individuals using the Sexual Relationships and Activities Questionnaire (SRA-Q). The SRA-Q was developed to assess the sexual activity of men and women in the English Longitudinal Study of Aging (ELSA; Lee et al., 2016). Discussion with data collectors and researchers living in the study area was conducted to confirm the contextual relevance and acceptability of questions. Following these steps, the SRA-Q was adapted with minor rephrasing to the local context and translated from English to French, before being translated verbally into Dioula or Mooré (the most frequently spoken local languages) by trained local fieldworkers, with data collected using tablet computers at the respondent place of residence. Translation practice was included in fieldworker training.

The SRA-Q asks 21 questions on sexual health and well-being including levels of sexual activity, sexual functioning, and concerns about sexual health (full questions are found in Table S1 in the supplemental materials). The SRA-Q includes 5 different domains, although analyses are only done for individual questions, following the methods from original publications (Lee et al., 2016).

1. Sexual behavior (3 questions) and activities (1 question) in the past 12 months
2. Sexual functioning during the past month (5 questions)
3. Changes in sexual behavior and function compared to a year ago (4 questions)
4. Sexual health concerns during the past month (4 questions)
5. Partnership and relationship satisfaction during the past 3 months (4 questions)

Response options used 5-point Likert scales, except for the count of sexual partners in the past 12 months which was a discrete numerical variable. All participants were asked 12 questions, with five additional sex-specific questions asked of women and four of men. Any sexual activity in the past year was defined as caressing, kissing or petting, foreplay, solitary or mutual masturbation, oral-genital sexual activities, and anal or vaginal intercourse.

### Procedure

The sample for the 2021 survey was drawn from the 2018 baseline study which has been described previously (Odland et al., 2020; Witham et al., 2019). Eligibility criteria included household residents aged 40 or older living in the Nouna HDSS area for at least the past six months, willingness to participate, and ability to give informed consent. Details for survey design including decisions around sample size and methods to reduce potential sources of bias have been described in detail in other publications (Goldberg et al., 2022; Odland et al., 2020).

### Statistical Analyses

Questions were analyzed separately rather than by domains due to the absence of previous psychometric testing. Likert scale responses were transformed to dichotomized variables according to methods used by Lee et al. (SRA-Q scoring is described in Table S1 in the supplemental materials) in their widely cited study utilizing the SRA-Q (Lee et al., 2016). The question reporting a discrete number of sexual partners in the last year was dichotomized to measure any or no sexual activity in the last year.

Sociodemographic and sexual health and well-being variables were described, with sexual health and well-being outcomes reported separately by sex and compared using the Chi-square test where the same questions were asked. Age (as a continuous variable), sex (for questions asked of men and women), and education were included in a multivariable logistic model. The overall levels of education were low in the population, so the analysis used any versus no education. Individuals with incomplete responses to any of the SRA-Q questions were excluded. Results were presented as adjusted odds ratios (aOR) and 95% confidence intervals (CI). All analyses were conducted using STATA/SE v17.0 (StataCorp, College Station, TX). We used the STROBE Checklist for Cross-Sectional Studies for reporting (Vandenbroucke et al., 2007).

## Results

### Demographics

Of 2336 included participants, 1328 (56.8%) were men, and 1008 (43.2%) were women (Table 1). The mean (standard deviation *(SD)*) age of men was 54.1(10.0) and women was 52.1 (8.8). One-fifth (19.9%) of participants were aged over 60, and half (49.3%) were aged between 40 and 50. Most individuals (81.5%) reported having no education, and a higher proportion of women (89.5%) reported no education than men (75.5%). Full sociodemographic details are in Table 1.

**Table 1:** Characteristics of the study sample included in the assessment of sexual health and well-being among married and cohabitating middle-aged and older people (n = 2336)

Characteristic	Total (*n* = 2336)	Men (*n* = 1328)	Women (*n* = 1008)
Age (in years) mean (*SD*)	53.2 (9.6)	54.1(10.0)	52.1 (8.8)
Age *n* (%)			
40 ≤ 50	1152 (49.3)	626 (47.1)	526 (52.1)
51 ≤ 60	718 (30.7)	403 (30.3)	315 (31.3)
61 ≤ 70	332 (14.2)	206 (15.5)	126 (12.5)
71+	134 (5.7)	93 (7.0)	41 (4.1)
Level of education* n* (%)			
No education	1905 (81.5)	1003 (75.5)	902 (89.5)
Any education	431 (18.5)	325 (24.5)	106 (10.5)

*SD* Standard deviation

### Sexual Behavior, Activities, and Functioning

Table 2 has the full results from the SRA-Q survey. In the domain of sexual behavior and activities, almost all individuals reported having a sexual partner in the past year (96.0% men, 93.2% women). Half of women (51.2%) versus 71.8% of men reported thinking about sex frequently (*p* < 0.001) and 69.4% of men compared to almost half of women (46.4%) reported frequent sexual activity (*p* < 0.001).

**Table 2:** Responses to questions in the 5 sexual health and well-being domains, overall and by sex

Sexual well-being domain and variables	Men *n* (%)	Women *n* (%)	Number (*n*)/total number responded^c^ (%)	*p* value
*Domain 1: Sexual behavior and activities*				
Any sexual partners in the past year	1276 (96.0%)	940 (93.2%)	2216/2336 (94.8%)	0.042
Thinking about sex frequently^a^	957 (71.8%)	517 (51.2%)	1474/2336 (63.1%)	< 0.001
Frequent sexual intercourse^a^	925 (69.4%)	468 (46.4%)	1393/2336 (59.6%)	< 0.001
Frequent kissing, fondling, or petting^a^	451 (33.8%)	236 (23.4%)	687/2336 (29.4%)	< 0.001
*Domain 2: Sexual functioning*^a^				
Erectile difficulties	340 (36.2%)	NA	340/939 (36.2%)	NA
Difficulty reaching ejaculation	187 (19.9%)	NA	187/939 (19.9%)	NA
Difficulty becoming sexually aroused	NA	353 (48.7%)	353/724 (48.7%)	NA
Dry vagina during sexual activity	NA	151 (20.9%)	151/724 (20.9%)	NA
Pain during/after sexual activity	NA	108 (14.9%)	108/724 (14.9%)	NA
*Domain 3: Changes in sexual behavior and function compared with a year ago*				
Decreased level of sexual drive/desire	624 (46.8%)	627 (62.1%)	1251/2336 (53.6%)	< 0.001
Decreased frequency of sexual activities	634 (47.6%)	638 (63.2%)	1272/2336 (54.4%)	< 0.001
Decreased ability to have an erection	407 (43.3%)	NA	407/939 (43.3%)	NA
Decreased ability to become sexually aroused	NA	457 (63.1%)	457/724 (63.1%)	NA
*Domain 4: Sexual health concerns *^a^				
Worried by level of sexual desire	479 (35.9%)	385 (38.2%)	864/2336 (37.0%)	0.292
Worried about the frequency of sexual activities	447 (33.7%)	364 (36.1%)	811/2336 (34.7%)	0.218
Concerned about ability to have an erection	309 (32.9%)	NA	309/939 (32.9%)	NA
Concerned about ability to become sexually aroused	NA	281 (38.8%)	281/724 (38.8%)	NA
*Domain 5: Partnership satisfaction *^b^				
Felt obligated to have sex	308 (23.1%)	281 (27.8%)	589/2336 (25.2%)	0.047
Did not feel emotionally close to partner during sex	525 (39.4%)	445 (44.1%)	970/2336 (41.5%)	< 0.001
Concerned about overall sex life	503 (37.7%)	437 (43.3%)	940/2336 (40.2%)	0.049
Dissatisfied with overall sex life	48 (3.6%)	68 (6.7%)	116/2336 (5.0%)	< 0.001

*NA* not applicable, *p* value using two sample test of proportions

^a^During the past 1 month

^b^During the past 3 months

^c^The denominator varies due to questionnaire routing based on sex

In the domain of sexual functioning (Table 2), one-third (36.2%) of men reported erectile difficulties, with one-fifth (19.9%) reporting difficulty reaching ejaculation in the past month. Half of women (48.7%) reported difficulty becoming sexually aroused in the past month, one-fifth (20.9%) reported a dry vagina during sexual activity, and 14.9% reported pain during or after sexual activity in the past month. Two-thirds (63.1%) of women reported a decrease in their ability to become sexually aroused compared to a year ago.

In the changes in sexual behavior and function domain (Table 2), two-thirds of women (62.1%) and half of men (46.8%) reported a decrease in their level of sexual drive/desire compared with a year ago. Similarly, two-thirds of women (63.2%) and half of men (47.6%) reported a decreased frequency of sexual activity compared with a year ago. Overall, women experienced a greater decrease in levels of sexual drive/desire and frequency of sexual activities within a year than men (*p* < 0.001 and *p* < 0.001 respectively). Compared to a year ago, 43.3% of men experienced a decrease in their ability to have an erection. Detailed response distributions for questions are reported in Table S1 in the supplemental materials.

### Sexual Health Concerns

In the domain of sexual health concerns, during the past month, over one-third of men (35.9%) and women (38.2%) were worried about sexual desire, and frequency of sexual activities (33.7% and 36.1% respectively). One-third of respondents reported physical challenges, with 32.9% of men concerned about their ability to have an erection during the past month and 38.8% of women concerned about their ability to become sexually aroused. Detailed response distributions for questions are reported in Table S1 in the supplemental materials.

### Partner Satisfaction

In the domain of partnership satisfaction, about one quarter reported feeling obligated to have sex, with lower rates among men (23.1%) compared to women (27.8%) (*p* = 0.047). A greater proportion of women (44.1%) did not feel emotionally close to their partner during sex than men (39.4%) (*p* < 0.001). Similarly, more women (43.3%) reported being concerned about overall sex life during the past three months compared to men (37.7%) (*p* = 0.049). However, very few men (3.6%) or women (6.7%) reported being dissatisfied with their overall sex life, with 71.0% of respondents reporting feeling moderately or very satisfied with their sex life. Detailed response distributions for questions are reported in Table S1 in the supplemental materials.

### Multivariable Analysis Results

Full results for the multivariable analyses are in Table 3.

**Table 3:** Binary logistic regression of dichotomized variables in the sexual relationships and activities domains

Sexual well-being domain and variable	Age (continuous)^a^		Sex: Women (versus men)		Any education (vs none)	
	aOR (95% CI)	*p* value	aOR (95% CI)	*p* value	aOR (95% CI)	*p* value
*Domain 1: Sexual behavior and activities*						
Sexual activity in the past year	1.00 (1.00, 1.00)	0.120	0.57 (0.39, 0.83)	< 0.001	1.04 (0.63, 1.74)	0.867
Thinking about sex frequently	0.93 (0.92, 0.94)	< 0.001	0.40 (0.33, 0.48)	< 0.001	1.50 (1.17, 1.93)	< 0.001
Frequent sexual intercourse	0.93 (0.92, 0.94)	< 0.001	0.37 (0.30, 0.44)	< 0.001	1.41 (1.11, 1.80)	0.012
Frequent kissing, fondling, or petting	0.96 (0.95, 0.97)	< 0.001	0.65 (0.54, 0.78)	< 0.001	2.00 (1.60, 2.49)	< 0.001
*Domain 2: Sexual functioning during the past month*						
Difficulty getting or keeping an erection that would be good enough for sexual activity [men only]	1.06 (1.04, 1.07)	< 0.001	NA	NA	0.60 (0.43, 0.83)	< 0.001
Difficulty reaching ejaculation [men only]	1.00 (1.00, 1.00)	0.891	NA	NA	1.17 (0.83, 1.66)	0.373
Difficulty feeling sexually aroused [women only]	1.07 (1.05, 1.09)	< 0.001	NA	NA	1.00 (0.64, 1.58)	0.989
Frequent uncomfortably dry vagina [women only]	0.97 (0.95, 0.99)	0.013	NA	NA	0.66 (0.37, 1.19)	0.174
Frequent pain or discomfort during/after sexual activity [women only]	0.97 (0.95, 1.00)	0.054	NA	NA	0.79 (0.41, 1.51)	0.438
*Domain 3: Changes in sexual behavior and function compared with a year ago*						
Decreased level of sexual drive/desire	1.10 (1.09, 1.11)	< 0.001	2.42 (2.01, 2.91)	< 0.001	0.96 (0.76, 1.21)	0.722
Decreased overall frequency of sexual activities	1.09 (1.08, 1.10)	< 0.001	2.04 (1.70, 2.44)	< 0.001	0.96 (0.76, 1.20)	0.711
Decreased ability to have an erection [men only]	1.00 (1.00, 1.01)	0.460	NA	NA	0.70 (0.53, 0.94)	0.023
Decreased ability to become sexually aroused [women only]	1.12 (1.09, 1.14)	< 0.001	NA	NA	1.12 (0.70, 1.82)	0.633
*Domain 4: Sexual health concerns during the past month*						
Worried or concerned about level of sexual desire	1.00 (1.00, 1.00)	0.988	1.09 (0.92, 1.30)	0.332	0.96 (0.77, 1.20)	0.754
Worried or concerned about the frequency of sexual activities	1.00 (1.00, 1.00)	0.983	1.11 (0.93, 1.33)	0.233	0.99 (0.79, 1.24)	0.933
Worried or concerned by ability to have an erection [men only]	1.00 (1.00, 1.00)	0.814	NA	NA	0.79 (0.58, 1.08)	0.144
Worried or concerned by current ability to become sexually aroused [women only]	1.01 (0.99, 1.03)	0.272	NA	NA	1.28 (0.82, 1.99)	0.277
*Domain 5: Partnership satisfaction during the past 3 months*						
Frequently had sex primarily because felt obliged to or it was a duty	0.96 (0.95, 0.97)	< 0.001	1.24 (1.02, 1.50)	0.033	1.19 (0.94, 1.52)	0.163
Frequently not feeling emotionally close to partner during sex	1.00 (1.00, 1.01)	0.355	1.18 (1.00, 1.40)	0.064	0.80 (0.65, 1.00)	0.052
Worry or concern about overall sex life	1.00 (1.00, 1.00)	0.823	1.24 (1.04, 1.46)	0.022	0.89 (0.72, 1.11)	0.301
Dissatisfaction with overall sex life	1.00 (1.00, 1.00)	0.303	1.99 (1.35, 2.93)	< 0.001	1.16 (0.70, 1.90)	0.568

*CI* confidence interval, *aOR* adjusted odds ratio, *NA* not applicable

^a^Change per each year older

#### Sexual Behavior, Activities and Functioning

The results of the binary logistic regression of dichotomized variables** (**multivariable analysis) are presented in Table 3. In the sexual behavior and activities domain, being women was associated with lower sexual activity in the last year (aOR = 0.57, 95% CI [0.39–0.83], *p* < 0.001) less frequent sexual intercourse (aOR = 0.37, 95% CI [0.30, 0.44], *p* < 0.001), or engaging in frequent kissing, fondly or petting (aOR = 0.65, 95% CI [0.54, 0.78], *p* < 0.001). Older age was not associated with differences in any sexual activity in the last year but was associated with a decrease in thinking about sex frequently (aOR = 0.93, 95% CI [0.92, 0.94], *p* < 0.001), frequent sexual intercourse (aOR = 0.93, 95% CI [0.92, 0.94], *p* < 0.001), and frequent kissing, fondling, or petting (aOR = 0.96, 95% CI [0.95, 0.97], *p* < 0.001). Having an education was associated with thinking about sex more frequently (aOR = 1.50, 95% CI [1.17, 1.93], *p* < 0.001), having more frequent intercourse (aOR = 1.41, 95% CI [1.11, 1.80], *p* = 0.012) and engaging in more frequent kissing, fondling, or petting (aOR = 2.00, 95% CI [1.60, 2.49], *p* < 0.001).

In the domain of sexual functioning, increased age was associated with men having slightly greater difficulty getting or keeping an erection (aOR = 1.06, 95% CI [1.04, 1.07] *p* < 0.001) and women having difficulty feeling sexually aroused (aOR = 1.07, 95% CI [1.05, 1.09], *p* < 0.001) but slightly less likely to report an uncomfortable dry vagina during sexual activity (aOR = 0.97, 95% CI [0.95, 0.99], p = 0.013). Men with any education were significantly less likely to experience difficulty getting or keeping an erection during the past month than men with no education (aOR = 0.60, 95% CI [0.43, 0.83], *p* < 0.001).

For changes in sexual behavior and function, women were more likely to report a decrease in sexual desire (aOR = 2.42, 95% CI [2.01, 2.91], *p* < 0.001) and overall frequency of sexual activities (aOR = 2.04, 95% CI [1.70, 2.44], *p* < 0.001) compared to men. Increased age was associated with decreased sexual desire in the past year (aOR = 1.10, 95% CI [1.09, 1.11], *p* < 0.001) and decreased frequency of sexual activities (aOR = 1.09, 95% CI [1.08, 1.10] *p* < 0.001). Older age was also associated with a decreased ability to become sexually aroused in women (aOR = 1.12, 95% CI [1.09, 1.14], *p* < 0.001).

#### Sexual Health Concerns

In the sexual health concerns domain, no significant associations were found between sexual health concerns in the past month and age, sex, or education.

#### Partnership Satisfaction

In the partnership satisfaction domain, women were more likely to frequently have sex because they felt obliged or duty-bound, (aOR = 1.24, 95% CI [1.02, 1.50], *p* = 0.033), be worried or concerned about their overall sex life (aOR = 1.24, 95% CI [1.04, 1.46] *p* = 0.022) and to have dissatisfaction with overall sex life (aOR = 1.99, 95% CI [1.35, 2.93], *p* < 0.001). Age was associated with a decrease in frequently having sex due to feeling obliged or duty-bound (aOR = 0.96, 95% CI [0.95, 0.97], *p* < 0.001).

## Discussion

Despite sexual health and well-being being recognized as an important measure of overall health and well-being, there has been limited research among older people in sub-Saharan Africa. We found that among married and cohabiting middle-aged and older adults in Burkina Faso, both sexes almost universally reported some sexual activity in the past year. We also found that issues with sexual health and well-being were common, including emotional disconnection during sex, feeling obligated to have sex, and sexual physical challenges such as difficulties with erection, ejaculation, dry vagina, and low sexual arousal. Approximately three-quarters of these adults reported being moderately or very satisfied with their sex life and a similar proportion were not at all or minimally worried about their overall sex life.

These findings of recent sexual activity are similar to other studies of middle-aged and older adults in a range of settings (Palacios-Ceña et al., 2012; Wang et al., 2015), including results from longitudinal data analysis of younger populations in four African countries which found that over 90% of respondents reported sexual activity during the past 12 months (Todd et al., 2009). However, the rates of sexual activity in this study were higher than those found in a study done in older adults in southwest Nigeria, although the population in the Nigerian study was older (mean age of 67 years) and included both ‘married’ and ‘non-married’ status (Egbewale & Adebimpe, 2020). In that study, adults who were living with a spouse were approximately six times more likely to be currently sexually active compared to those not living with a spouse.

The levels of overall sexual dysfunction found in this study were slightly lower than those in a population of people with diabetes in Ethiopia (Asefa et al., 2019), which found that the most commonly reported type of sexual dysfunction in both sexes was a reduction in sexual desire with rates similar to those found in our cohort. Notably, the study in Ethiopia utilized a different self-report tool; the Changes in Sexual Functioning Questionnaire-14 items (CSFQ-14). In contrast, there were higher rates of erectile dysfunction and lower rates of being worried by levels of sexual desire or activities in our study compared to those of an aging cohort from the United Kingdom which used the same self-report questionnaire tool (Lee et al., 2016).

Sex differences were seen across several survey domains. Men were more likely to report frequently engaging in sexual activities and frequently thinking about sex compared to women. When compared to men, women in our study had lower self-ratings of sexual health and well-being across a number of domains. These included lower levels of partner satisfaction, being less likely to feel emotionally close to partners during sex, and more likely to frequently have sex primarily because they felt obliged or duty-bound, with higher dissatisfaction with their overall sex life. This is in contrast to findings in the UK, where reported concerns about or dissatisfaction with overall sex life were higher in men aged 50 and older compared to women (Lee et al., 2016). These differences highlight the need to further explore sexual health and well-being within the context of cultural and societal norms and expectations of sexual activity and sexuality in middle-aged and older adults in sub-Saharan Africa, acknowledging that findings from high income country settings do not directly transfer to different contexts.

Our study found that, regardless of sex, older age was associated with a decrease in several measures of sexual health and well-being including sexual activity, thoughts about sex, and frequency of sexual intercourse, kissing, fondling, or petting. These results are similar to studies in the Netherlands, Korea, United States, and Morocco which also found that aging was associated with increased sexual dysfunction and less sexual activity (Berrada et al., 2003; Kleinstäuber, 2017; Wang et al., 2015). We found a slight decline in discomfort during/after sexual activity among women as age increased, which corresponds to findings from the United Kingdom ELSA study (Lee et al., 2016). These changes underscore the importance of ongoing assessment by primary care providers as individuals age to understand sexual and well-being and inform culturally appropriate interventions to address changing sexual health and well-being concerns and function in both sexes. The growing strategies of task shifting to community health workers and trained peers could offer opportunities in countries in sub-Saharan Africa for expanding the dialogue around sexual health and well-being and increasing availability of potential lifestyle and behavioral interventions to improve or maintain sexual health and well-being in middle-aged and older adults.

Of note, older age was determined to be greater than 40 years in this study. Life expectancy in countries with lower sociodemographic indices are on average about 20 years lower compared to high-income countries (Zheng & Canudas-Romo, 2024). Life expectancy in Burkina Faso in 2021 was 62.3 years (World Health Organization, n.d.-b), compared to 80.1 years in the UK (World Health Organization, n.d.-c), where the SRA-Q survey was developed. It is difficult to determine whether aging is interpreted similarly in different cultures. Evidence suggests that compared to Western countries, the lower life expectancy in Burkina Faso means that a different scaling of when “older age” starts is likely to exist and that in Burkina Faso “old” is more likely to be regarded in terms of cultural and physiological determinants than by an age threshold (Bezzina, 2020; Schönstein et al., 2023; Teuscher, 2009). Work with older people in other countries in Sub-Saharan Africa (Uwizeyimana et al., 2024) and elsewhere (Casadei Donatelli et al., 2024; Usmani et al., 2024) corroborates this hypothesis. A study looking at subjective age in adults aged 40 and older in Burkina Faso shows that adults felt less young than their chronological age compared to similar age groups in Western studies including countries such as the United States, United Kingdom, and Denmark (Schönstein et al., 2021). Whilst our study solely looked at biological age, rather than chronological age, it may be important to consider whether what is considered older age differs within LMICs.

Due to decisions based on advice around cultural considerations, this study only assessed the sexual activity and behaviors of adults aged 40 and over who were married or cohabitating. The literature suggests that there is an increased prevalence of sexual activity among middle-aged and older adults who have a spousal or long-term romantic partner (Hyde et al., 2010; Lindau et al., 2007; Negin et al., 2016), compared to adults who do not. A 2024 scoping review identified eight studies looking at sexual well-being specifically among community samples of partnered adults and couples aged over 60, although none were conducted in Sub-Saharan Africa (Bigras et al., 2024). Results showed that although physical and psychological health were positively associated with sexual function, factors such as relationship satisfaction and intimacy were key correlates of sexual well-being (sexual function, satisfaction, and distress) among middle-aged and older adults involved in a relationship and among couples (Bigras et al., 2024). Our study population was limited to married or cohabiting individuals, representing the results of a population group known to have a higher predicted sexual activity. This study may therefore add value in exploring the sexual well-being of partnered individuals engaged in a long-term spousal or romantic relationship with limitations in the generalizability of its findings to populations without long-term married or cohabitating partnerships.

### Limitations

Our study had several limitations. First, The SRA-Q tool was developed in the United Kingdom and whilst undergoing a translation process from English to French with some adaptation and then into local spoken languages, it has not been formally validated for use in this study setting, introducing translation and cultural biases. Ideally, a pilot study or cognitive interviews with the target population would have strengthened the study’s reliability and validity, however, were not able to be conducted, and should be considered for future work. In addition, this study dichotomized ordinal or continuous variables according to previous use of the SRA-Q, which could impact the robustness of the study’s findings through loss of information and statistical power. Additionally, as noted above based on advice from the local study team about local sensitivities, sexual and well-being questions were only asked of married or cohabitating individuals. Since sexual activity, behaviors, and concerns may vary based on relationship status, the generalizability of our findings to the wider population including unmarried or single middle-aged and older adults is limited. Also while the SRA-Q survey focuses on both biological and behavioral aspects of sexual well-being, it does not measure other psychological, social or cultural dimensions of sexual well-being (Syme et al., 2019). The survey also did not ask about menopause status, so its association with sexual health and well-being outcomes among women was not explored. Finally, we did multiple comparisons and chose to not use the Bonferroni or other correction reflecting other analyses of the Nouna survey. Work to do psychometrics testing through confirmatory factor analysis on the grouping of the individual questions within a category is ongoing.

### Conclusions

This study adds to the growing body of research examining sexual health and well-being in married and cohabitating middle-aged and older adults in Sub-Saharan Africa and other LMIC settings. Further research is needed to better understand the complexity of associations between socio-cultural, psychosocial, and health-related variables on sexual health and well-being in middle-aged and older adults, and to develop and test context-specific interventions to improve sexual health and well-being in Burkina Faso and the region.

## Supplementary Information

Below is the link to the electronic supplementary material.

Supplementary file1 (DOCX 137 KB)

## Data Availability

Data are available from Dr. Davies upon reasonable request.
