# Supplementary material for: A Cross-Sectional Study of Sexual Health and Well-Being in Married or Cohabitating Middle-Aged and Older Adults in Nouna, Burkina Faso
Source: Arch Sex Behav. 2025 Sep 30;54(9):3541–50. doi: 10.1007/s10508-025-03223-1 (PMC12675568; doi:10.1007/s10508-025-03223-1)
Supplement: Supplementary file 1 — Supplementary file1 (DOCX 137 KB) [file 10508_2025_3223_MOESM1_ESM.docx]

**Figure S1**

*Sexual Health and Well-being in Middle-aged and Older Adults in Nouna, Burkin Faso CONSORT Diagram*


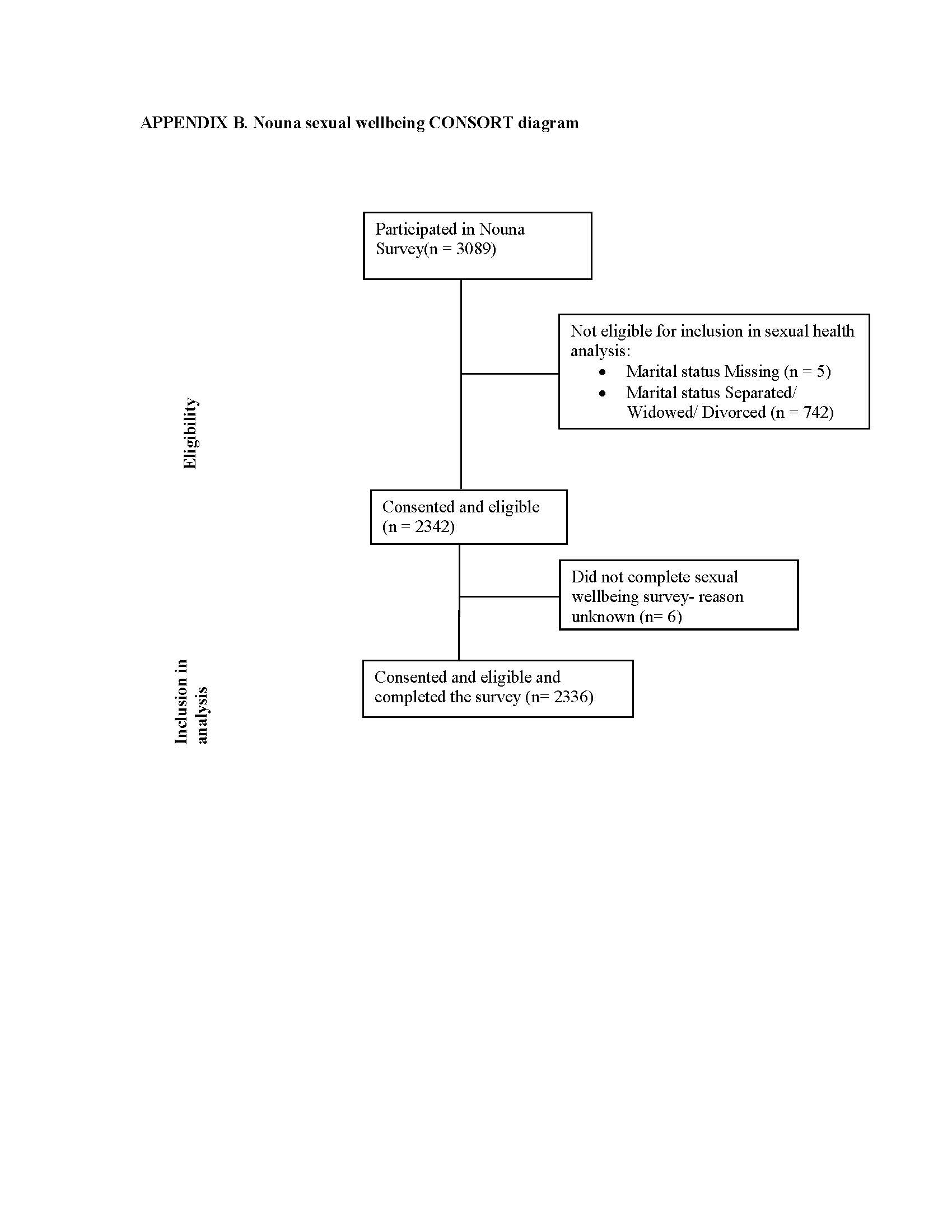


**Table S1**

*Sexual Relationships and Activities Questionnaire (SRA-Q) 21 Questions by Participant Likert Response Distribution and Description for Dichotomization for Multivariable Analysis. Questions Asked only of Men or Women are Labeled.*

| Sexual Relationships and Activities Questionnaire (SRA-Q) Participant Response Distribution | | | | | | | | |
| --- | --- | --- | --- | --- | --- | --- | --- | --- |
| Domain | Question | Response set and distribution *n* (%) | | | | | | Binary sexual well-being variable |
| Sexual behavior and activities during the past month (*n* = 2336) | How often did you think about sex? | Never | Seldom | | Sometimes | Frequently | Always | Never/ seldom (0), Sometimes/ Frequently/ Always (1) |
|  |  | 205 (8.78) | 657 (28.13) | | 1122 (48.03) | 334 (14.3) | 18 (0.77) |  |
|  | How many times have you had or attempted sexual intercourse? | 234 (10.02) | 709 (30.35) | | 1101 (47.13) | 288 (12.3) | 4 (0.17) |  |
|  | How frequently did you engage in other sexual activities (kissing, petting, or fondling)? | 871 (37.29) | 778 (33.3) | | 590 (25.26) | 94 (4.02) | 3 (0.13) |  |
|  | How many sexual partners did you have in the past 12 months? (measure of sexual activity in last year; continuous number) | 0 partners | | 1 partner | | ≥2 partners | | 0 sexual partners (0), 1 or more sexual partners (1) |
|  |  | 120  (5.14) | | 2179 (93.28) | | 37 (1.58) | |  |
| Sexual functioning during the past month | How often did you feel sexually aroused during sexual activity? [women] (*n* = 724) | Never | Seldom | | Sometimes | Frequently | Always | Always/Frequently/Sometimes (0), Seldom/Never (1) |
|  |  | 107 (14.78) | 246 (33.98) | | 293 (40.47) | 77 (10.64) | 1 (0.14) |  |
|  | How often did you have an uncomfortably dry vagina during sexual activity? [women]  (*n* = 724) | 270 (37.29) | 303 (41.85) | | 130 (17.96) | 20 (2.76) | 1 (0.14) | Never/ seldom (0), Sometimes/ Frequently/ Always (1) |
|  | How often did you experience pain or discomfort during/after sexual activity? [women]  (*n* = 724) | 254 (35.08) | 362 (50) | | 101 (13.95) | 5 (0.69) | 2 (0.28) |  |
|  | Are you able to get or keep an erection which would be good enough for sexual activity? [men]  (*n* = 939) | 101 (10.76) | 239 (25.45) | | 369 (39.3) | 228 (24.28) | 2 (0.21) | Always/Frequently/Sometimes (0), Seldom/Never (1) |
|  | When you had sexual stimulation how difficult was it for you to reach ejaculation? [men]  (*n* = 939) | None | Mild | | Moderate | Severe | Impossible | Mild/ None (0), Impossible/ Severe/ Moderate (1) |
|  |  | 492 (52.4) | 260 (27.69) | | 151 (16.08) | 20 (2.13) | 16 (1.7) |  |
| Changes in sexual behavior and function compared with a year ago | Has your sexual desire/drive changed?  (*n* = 2336) | Increased a lot | Increased a little | | Neither increased or decreased | Decreased a little | Decreased a lot | Not increased/ decreased/ Increased a little/ Increased a lot (0), Decreased a lot/ Decreased a little (1) |
|  |  | 8 (0.34) | 38 (1.63) | | 1039 (44.48) | 799 (34.2) | 452 (19.35) |  |
|  | Has the overall frequency of your sexual activities changed?  (*n* = 2336) | 7 (0.3) | 31 (1.33) | | 1026 (43.92) | 802 (34.33) | 470 (20.12) |  |
|  | Has your ability to become sexually aroused changed? [women]  (*n* = 724) | 1 (0.14) | 11 (1.52) | | 255 (35.22) | 280 (38.67) | 177 (24.45) |  |
|  | Has your ability to have an erection changed? [men]  (*n* = 939) | 3 (0.32) | 25 (2.66) | | 504 (53.67) | 303 (32.27) | 104 (11.08) |  |
| Sexual health concerns during the past month | Have you been worried or concerned by your level of sexual desire?  (*n* = 2336) | Not at all worried | A little worried | | Moderately worried | Very much worried | Extremely worried | Not at all worried (0), Extremely worried/ Very much worried, Moderately worried, A little worried (1) |
|  |  | 1472 (63.01) | 352 (15.07) | | 380 (16.27) | 61 (2.61) | 71 (3.04) |  |
|  | Have you been worried or concerned by the frequency of your sexual activities?  (*n* = 2336) | 1525 (65.28) | 346 (14.81) | | 360 (15.41) | 41 (1.76) | 64 (2.74) |  |
|  | Are you worried or concerned by your current ability to become sexually aroused? [women]  (*n* = 724) | 443 (61.19) | 132 (18.23) | | 104 (14.36) | 14 (1.93) | 31 (4.28) |  |
|  | Have you been worried or concerned by your ability to have an erection? [men]  (*n* = 939) | 630 (67.09) | 141 (15.02) | | 134 (14.27) | 15 (1.6) | 19 (2.02) |  |
| Partnership satisfaction during the past 3 months | How often did you have sex primarily because you felt obliged to or it was your duty?  (*n* = 2336) | Never | Seldom | | Sometimes | Frequently | Always | Never/ Seldom (0), Sometimes/ frequently/Always (1) |
|  |  | 967 (41.4) | 780 (33.39) | | 258 (11.04) | 312 (13.36) | 19 (0.81) |  |
|  | How often did you feel emotionally close to your partner when you had sex?  (*n* = 2336) | 159 (6.81) | 814 (34.85) | | 393 (16.82) | 563 (24.1) | 407 (17.42) |  |
|  | How worried or concerned have you been about your overall sex life?  (*n* = 2336) | Not at all worried | A little worried | | Moderately worried | Very much worried | Extremely worried | Not at all worried (0) Extremely worried/ Very much worried/ Moderately worried/ A little worried (1) |
|  |  | 1396(59.76) | 471(20.16) | | 316(13.53) | 99(4.24) | 54(2.31) |  |
|  | How satisfied have you been with your overall sex life?  (*n* = 2336) | Very satisfied | Moderately satisfied | | Neither satisfied nor dissatisfied | Moderately dissatisfied | Very dissatisfied | Neither satisfied nor dissatisfied/ Moderately satisfied/ Very satisfied (0), Very dissatisfied/ Moderately dissatisfied (1) |
|  |  | 85 (3.64) | 1574 (67.38) | | 561 (24.02) | 99 (4.24) | 17 (0.73) |  |
